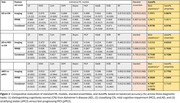# AutoML‐Multiverse: Model Agnostic Framework for Optimizing Individual‐level Alzheimer's Disease Classification

**DOI:** 10.1002/alz70856_102543

**Published:** 2025-12-25

**Authors:** Maitrei Kohli, Pedro da Costa, Robert Leech, James H. Cole

**Affiliations:** ^1^ UCL Hawkes Institute, University College London, London, United Kingdom; ^2^ Institute of Psychiatry, Psychology and Neuroscience, King's College London, London, United Kingdom

## Abstract

**Background:**

Machine learning (ML) methods are widely used to develop predictive models for Alzheimer's disease (AD) stage classification, crucial for patient stratification in clinical trials. While the goals of these models are clear, the analytic processing and modelling details are not, causing conflicting results, and limiting clinical utility. Here, we propose a novel automated ML (autoML) approach for predictive classification of patient‐level AD stage to mitigate experimenter bias, eliminate arbitrary decisions, and enhance predictive accuracy for clinically significant outcomes.

**Method:**

Our *AutoML‐multiverse* framework navigates 20,000 ML pipelines using Bayesian Optimization in a low‐dimensional configuration space for efficient sampling. Further, it constructs data‐driven ensembles by integrating complementary information from diverse pipelines into stacked models, boosting predictive performance.

Our primary goal was classification into three diagnostic categories: Task1) cognitively normal (CN) versus AD, Task2) AD versus mild cognitive impairment (MCI) versus CN, and Task3) stable MCI (sMCI) versus progressive MCI (pMCI). We benchmarked our AutoML‐Multiverse framework against nine standalone ML models and their stacked ensemble across three settings: i) structural MRI data, ii) clinical parameters (age, sex, MMSE), and iii) structural MRI plus clinical parameters.

Regional volumes from T1‐weighted MRI scans (ICV‐corrected ventricle, hippocampus, whole‐brain, entorhinal, fusiform, mid‐temporal) and clinical data from the ADNI dataset were standard‐scaled. We included n = 606 (303 CN, 303 AD) for Task1, n = 1930 (760 CN, 867 MCI, 303 AD) for Task2, and n = 369 (224 sMCI, 145 pMCI) for Task3.

**Result:**

Figure 1 shows that standalone ML models had limited predictive performance, while stacked ensembles offering marginal gains. In contrast, AutoML‐derived ensembles consistently delivered superior accuracy across all diagnostic tasks. Notably, in Task3, distinguishing sMCI from pMCI, the AutoML ensemble achieved 77.55% balanced accuracy, highlighting the clinical relevance of MRI data for precise patient stratification.

**Conclusion:**

Preliminary findings reveal three key strengths of the AutoML‐multiverse framework: (1) superior predictive performance over individual pipelines, (2) data‐driven ensemble construction that reduces biases and arbitrary decisions, and (3) clinically relevant integration of neuroimaging, particularly for distinguishing slow from fast progressors, with potential utility in clinical trials. Future work will validate the robustness and generalisability of these results.